# Identification of TgCBAP, a Novel Cytoskeletal Protein that Localizes to Three Distinct Subcompartments of the *Toxoplasma gondii* Pellicle

**DOI:** 10.1371/journal.pone.0098492

**Published:** 2014-06-02

**Authors:** Lucas D. Tilley, Shruthi Krishnamurthy, Nicholas J. Westwood, Gary E. Ward

**Affiliations:** 1 Department of Microbiology and Molecular Genetics, University of Vermont, Burlington, Vermont, United States of America; 2 School of Chemistry and Biomedical Sciences Research Complex, University of St Andrews and EaStCHEM, North Haugh, St Andrews, Fife, Scotland, United Kingdom; Univ. Georgia, United States of America

## Abstract

The cytoskeletons of *Toxoplasma gondii* and related apicomplexan parasites are highly polarized, with apical and basal regions comprised of distinct protein complexes. Components of these complexes are known to play important roles in parasite shape, cell division, and host cell invasion. During an effort to discover the biologically relevant target of a small-molecule inhibitor of *T. gondii* invasion (Conoidin A), we discovered a novel cytoskeletal protein that we named TgCBAP (Conserved Basal Apical Peripheral protein). Orthologs of *TgCBAP* are only found in the genomes of other apicomplexans; they contain no identifiable domains or motifs and their function(s) is unknown. As a first step toward elucidating the function of this highly conserved family of proteins, we disrupted the *TgCBAP* gene by double homologous recombination. Parasites lacking TgCBAP are as sensitive to the effects of Conoidin A as wild-type parasites, demonstrating that TgCBAP is not the biologically relevant target of Conoidin A. However, *ΔTgCBAP* parasites are significantly shorter than wild-type parasites and have a growth defect in culture. Furthermore, TgCBAP has an unusual subcellular localization, forming small rings at the apical and basal ends of the parasite and localizing to punctate, ring-like structures around the parasite periphery. These data identify a new marker of the apical and basal subcompartments of *T. gondii*, reveal a potentially novel compartment along the parasite periphery, and identify TgCBAP as a determinant of parasite size that is required for a maximally efficient lytic cycle.

## Introduction

*Toxoplasma gondii* is a member of the Phylum Apicomplexa, which includes many organisms pathogenic to humans (*e.g*., *Plasmodium*, *Cryptosporidium* and *Babesia spp*.), domestic animals and wildlife (*e.g*., *Eimeria, Sarcocystis*, and *Theileria spp*.). Infections by apicomplexan parasites can cause severe disease in the host, often resulting in significant morbidity, mortality, and socio-economic burden. Knowledge of the basic biology of these evolutionarily divergent parasites is a prerequisite for designing effective diagnostics and therapeutics. *T. gondii* is considered a model apicomplexan parasite due to the relative ease with which it can be propagated and the availability of powerful molecular genetic tools to study conserved features and mechanisms of apicomplexan biology [1]–[6].

Apicomplexan parasites share a unique cytoskeletal and pellicular architecture [7]. They are bounded by an outer plasma membrane supported by flattened, plate-like alveoli termed the inner membrane complex (IMC) [8] [9]; together, the plasma membrane and IMC comprise the pellicle. The subpellicular network is a meshwork of intermediate-filament like proteins located on the interior surface of the IMC [10]–[12]. This structure acts as a membrane skeleton providing mechanical strength to the pellicle [13]. Adjacent to the cytoplasmic face of the *T. gondii* subpellicular network, 22 highly stable microtubules spiral posteriorly from an apical polar ring, terminating approximately two-thirds down the length of the parasite [14]. At the most apical end of the pellicle, *T. gondii* and many other apicomplexans are equipped with a dynamic spring-like structure called the conoid. The conoid is composed of a novel polymeric form of tubulin and assumes the shape of a truncated cone that extends and retracts as the parasites glide along surfaces and during invasion of host cells [15]. The function of the conoid is currently unknown.

In the course of developing a set of chemical tools to study *T. gondii* motility and invasion, a small molecule referred to as Conoidin A [2,3-bis(bromomethyl)quinoxaline 1,4-dioxide] was found to be an inhibitor of Ca^2+^-induced conoid extension and host cell invasion [16]. A subsequent report identified peroxiredoxin II (TgPRXII) and a protein we refer to here as TgCBAP (for Conserved Basal Apical Peripheral protein) (TgME49_267500) as Conoidin A-binding proteins in *T. gondii* [17]. Conoidin A was shown to inhibit recombinant TgPRXII through covalent modification of the peroxidatic cysteine, but *ΔTgPRXII* knockout parasites did not show reduced sensitivity to Conoidin A, arguing that TgPRXII is not the biologically relevant target of the compound [17].

We report here the generation of a *TgCBAP* knockout parasite (Δ*TgCBAP*), which also showed no change in sensitivity to Conoidin A. However, Δ*TgCBAP* parasites have an altered morphology compared to wild-type parasites and show a fitness defect in culture. TgCBAP has an extraction profile that suggests a cytoskeletal association, and an unusual localization within the parasite, forming both apical and basal ring structures and distinct punctate spots along the parasite periphery. TgCBAP is therefore a previously unrecognized pellicular protein of apicomplexan parasites that plays a role in regulating parasite size and fitness.

## Materials and Methods

### Culture of parasites

*T. gondii* tachyzoites (*ku80::hxgprt* strain [18] kindly provided by Dr. Vern Carruthers, University of Michigan) and transgenic derivatives were used for all experiments unless otherwise indicated. Parasites were serially passaged in confluent human foreskin fibroblasts (HFFs; ATCC CRL-1634) (37°C, 5% CO_2_, constant humidity) in Dulbecco's Modified Eagle's Medium (DMEM) containing 1% (v/v) fetal bovine serum (FBS), 10 µg/ml streptomycin sulfate, 10 units/ml penicillin G, and 10 mM HEPES buffer, pH 7.2 [4].

### Production of TgCBAP rabbit antiserum and blot affinity purification

The open reading frame encoding TgCBAP was amplified from cDNA using primers designed to add six tandem histidine residues (6X His-tag) to the N-terminus of TgCBAP. The N-terminal 6X His-TgCBAP fusion amplicon was ligated into pET28a(+) (Novagen, EMD Millipore, Darmstadt, Germany). The resulting construct (pET28a-NHis-TgCBAP) was used to transform *E. coli* BL21(DE3)RIPL cells (Agilent Technologies, Santa Clara, CA). Production of NHis-TgCBAP was induced in mid-log phase cultures by the addition of 0.4 mM isopropyl β-D-1-thiogalactopyranoside (IPTG) and continued incubation at 37°C for 4 h. Bacterial cells were harvested by centrifugation (4,000*xg*) for 10 min at 4°C. The resulting cell pellets were lysed using Bacterial Protein Extraction Reagent (B-PER; Pierce, Rockford, IL) according to the manufacturer's instructions. Lysates were centrifuged at 21,000*xg* for 30 min at 4°C to separate inclusion bodies containing NHis-TgCBAP from soluble protein. Inclusion bodies were solubilized by boiling in 1X Laemmli sample buffer (50 mM Tris-HCl, pH 6.8, 2% (w/v) SDS, 10% (v/v) glycerol, 1% (v/v) β-mercaptoethanol, 12.5 mM EDTA, 0.02% (w/v) bromophenol blue) and resolved using 10% polyacrylamide-SDS gels. Gels were stained with Colloidal Coomassie Blue G-250. Gel slices containing a total of ∼500 µg of NHis-TgCBAP protein were submitted to Cocalico Biologicals, Inc. (Reamstown, PA) for antiserum generation in rabbits pre-screened for low pre-bleed serum reactivity toward *Toxoplasma* antigens.

Procedures for the blot-affinity purification of the TgCBAP antibody were adapted from [19] and [20]. *E. coli* lysates containing recombinant NHis-TgCBAP were transferred to a PVDF membrane and blocked in 5% (w/v) nonfat milk in TBS-T (20 mM Tris pH 7.4, 150 mM NaCl, 0.1% (v/v) Tween) for 1 h. TgCBAP rabbit antiserum (diluted 1∶10 in TBS-T+5% nonfat milk) was added to the membrane and incubated 18–20 h at 4°C. The diluted antiserum was removed and a fresh volume of diluted antiserum was added to the membrane two more times to increase the total amount of antibody bound. Each volume of diluted antiserum was checked before and after incubation for reactivity toward TgCBAP by Western blot using *ku80::hxgprt* and Δ*TgCBAP* lysates. After the third incubation, the membrane was washed three times in TBS-T for 15 min, once in PBS for 15 min, and once in deionized water for 15 min at 23°C. A strip of membrane containing TgCBAP was excised, cut into small pieces, and placed into a 1.5 ml microcentrifuge tube. The absorbed TgCBAP antibody was eluted 3 times by incubation of the membrane pieces with 200 µl of 0.2 M glycine, pH 2.5 for 3 min. Each elution was immediately neutralized by addition of 20 µl 1 M Tris, pH 8.0. Elutions were combined and the antibody solution was concentrated to 50 µl using an Amicon Ultra 0.5 ml centrifugal filter (MWC 10 kDa; Millipore, EMD Millipore, Darmstadt, Germany). The purified antibody was stored at 4°C.

### Generation of the TgCBAP knockout parasite line (Δ*TgCBAP*)

All PCR reactions were performed with Phusion DNA polymerase (New England Biolabs, Ipswitch, MA). All primers were synthesized by Sigma-Aldrich (The Woodlands, TX) and restriction enzymes were sourced from NEB. PCR was performed on *ku80::hxgprt* genomic DNA using primers KpnITgCBAP5-F (5′-AAAAGGTACCTGGCTTGGAATGAGTGTGAATG-3′) and Hind3TgCBAP5-R (5′-GGGGAAGCTTAACTCGTCAACCGACAATTGCC-3′), and BamHITgCBAP3-F (5′-AAAAGGATCCGTAGAAGCTCCAAGAGCCGTTG-3′) and XbaITgCBAP3-R (5′- GGGGTCTAGATCTCTTGTCAACACGCCATGGG-3′) to amplify the 5′ and 3′ flanking regions of *TgCBAP*, respectively. To generate the Δ*TgCBAP* knockout plasmid, the 0.95 kb PCR amplicon of the 5′ flanking region was cloned into the KpnI and HindIII sites upstream of the *GRA1* promoter and the 0.8 kb amplicon of the 3′ flanking region was cloned into the BamHI and XbaI sites downstream of the *SAG1* 3′ UTR in pGRA1/ble [21]. The plasmid was linearized with KpnI prior to transfection of *ku80::hxgprt* parasites. The transfected parasites were selected with phleomycin as previously described [21]. Clones lacking TgCBAP were isolated by limiting dilution, identified by immunofluorescence and verified by Western blot using TgCBAP rabbit antiserum.

### Endogenous locus tagging of TgCBAP with 3xmyc or YFP

*T. gondii ku80::hxgprt* genomic DNA was used as a template to amplify a 1.26 kb genomic fragment of the *TgCBAP* coding region using primers PacITgCBAPg-F (5′-CCCCTTAATTAAGCAGCTCTTCGCTCGAATGCAGCAAG-3′) and AvrIITgCBAPg-R (5′-GGGGCCTAGGCTGGTAGACGGGACTGTAAAGATTTG-3′) or EcoRITgCBAPg-F (5′-GGGGGAATTCCTGGTAGACGGGACTGTAAAGATTTG-3′). The genomic fragments were cloned into either the PacI and AvrII sites of p3xmyc-dhfr or the PacI and EcoRI sites of pYFP-hxgprt, fusing the penultimate *TgCBAP* codon in-frame with either the 3xmyc or YFP coding sequence. Plasmids were linearized with BsiWI prior to transfection of Δ*ku80* parasites [22], kindly provided by Dr. David Bzik, Dartmouth College. Parasites transfected with p*TgCBAP*-3xmyc-dhfr were selected with pyrimethamine as previously described [4]. Clones expressing TgCBAP-3xmyc were isolated by limiting dilution, then identified by immunofluorescence and verified by Western blot using a mouse monoclonal anti-Myc antibody (9E10; Developmental Studies Hybridoma Bank, University of Iowa). Transient p*TgCBAP*-YFP-HXGPRT transfectants were identified by live-cell immunofluorescence.

### Complementation of the ΔTgCBAP parasite line (Δ*TgCBAP*::TOXOF64)

To complement the deletion of *TgCBAP*, the cosmid TOXOF64 containing the entire *TgCBAP* locus was electroporated into Δ*TgCBAP* parasites. Transfectants were selected using pyrimethamine as previously described [4]. Complemented parasites expressing TgCBAP were identified by Western blot using TgCBAP rabbit antiserum and by immunofluorescence using blot-affinity purified TgCBAP polyclonal antibody.

### Immunofluorescence

Extracellular parasites were attached to glass coverslips using BD Cell-Tak (BD Bioscience, San Jose CA). All manipulations were carried out at 23°C. For labeling with blot-affinity purified anti-TgCBAP antibody, attached parasites were extracted in 0.5 mM sodium deoxycholate in PBS for 5 min, fixed in PBS containing 4% (v/v) paraformaldehyde for 10 min, and then blocked in PBS containing 5% (w/v) nonfat milk for 30 min. The parasites were incubated with blot-affinity purified anti-TgCBAP diluted 1∶200 in PBS+5% nonfat milk for 30 min. Parasites were similarly labeled with either mouse MAb 45.36 anti-IMC1 (1∶2000), rabbit anti-TgMORN1 (1∶1000; a generous gift from Dr. Marc-Jan Gubbels; [23]), or mouse anti-ISP1 (1∶2000; a generous gift from Dr. Peter Bradley; [24]). Parasites labeled with anti-α-tubulin antibody B-5-1-2 (Sigma-Aldrich, The Woodlands, TX) were first adhered to BD CellTak-coated coverslips and extracted in 10 mM deoxycholate in PBS for 30 min, fixed in PBS containing 4% (v/v) paraformaldehyde for 10 min, blocked with PBS containing 2% (w/v) bovine serum albumin (BSA), and labeled with MAb B-5-1-2, diluted 1∶1000 in PBS+2% (w/v) BSA. After incubation with primary antibodies, the samples were incubated with a 1∶1000 dilution of Alexa488- and/or Alexa546-conjugated secondary antibody (Invitrogen, Carlsbad, CA). Fluorescently-stained parasites were imaged using a 60X PlanApo objective on Nikon Eclipse TE300 epifluorescence microscope. Images were captured using an iXon 885 EMCCD camera (Andor Technology, Belfast, Ireland) cooled to −70°C and driven by NIS Elements v. 3.20 software (Nikon Instruments). Final images were processed by Adobe Photoshop. Deconvolved images were captured using a Nikon Eclipse Ti2000 microscope with a PlanApo 60X (1.4 NA) oil immersion objective (Olympus, Melville, NY), High-Q filter sets (Chroma Technology, Brattleboro,VT) a Z-spacing of 0.26 µm, and a Qimaging ExiBlue digital camera (Surrey, British Colombia, Canada). Image deconvolution parameters were set for 40 iterations and deconvolved using Autoquant X software (MediaCybernetics).

### Conoid extension assay

Conoid extension assays were performed as previously described [25]. Syringe-released and filtered parasites were pretreated for 15 min with 5 µM Conoidin A, 50 µM Conoidin A, or an equivalent amount of DMSO, then incubated for 5 min at 23°C in Hanks Buffered Salt Solution (HBSS; Invitrogen) containing 10 µM ionomycin (Sigma) or an equivalent volume of DMSO. Parasites were fixed for 1 h at 23°C in HBSS containing 1% (v/v) glutaraldehyde, smeared onto a glass slide, and air-dried. 100 parasites were scored as exhibiting either an extended or retracted conoid by phase contrast microscopy. Conoid extension assays were performed three times for total of 300 parasites scored for each treatment.

### Differential detergent extraction of extracellular parasites

Syringe-released, filtered parasites were resuspended at 1×10^8^ parasites/ml and extracted for 30 min on ice using the following detergents: Digitonin (0.015% [v/v] digitonin, 150 mM NaCl, 1X PBS, pH 7.4); Triton X-100 (1.5% [v/v] Triton X-100, 150 mM NaCl, 1X PBS, pH 7.4); RIPA buffer (1% [v/v] Triton X-100, 0.5% [w/v] sodium deoxycholate, 0.1% [v/v] SDS, 50 mM Tris, pH 8.0, 150 mM NaCl); or SDS (1% [v/v] SDS, 50 mM Tris, pH 8.0, 150 mM NaCl). Extracts were then centrifuged (21,000*xg*) for 30 min at 4°C to separate soluble and insoluble cellular components. Equivalent amounts of protein from supernatants and pellets were solubilized by boiling in 1X Laemmli sample buffer. Samples were separated through 12% polyacrylamide-SDS gels, transferred to Immobilon-FL membranes (Millipore), blocked with 5% [w/v] nonfat milk, then probed with anti-TgCBAP rabbit antiserum (1∶100), MAb 45-36 (anti-IMC1, 1∶1000), MAb B-5-1-2 (anti-α-tubulin, 1∶400), and anti-TgACT1 (anti-actin, 1∶10,000; a generous gift from Dr. David Sibley, Washington University in St. Louis). Membranes were washed in PBS, incubated for 2 h with secondary antibodies (IRDye 800 CW-conjugated anti-mouse and IRDye 680 CS-conjugated anti-rabbit, each at 1∶20,000), washed in PBS, and scanned using an Odyssey Imaging System CLx (LI-COR Biosciences, Lincoln, NE).

### Growth competition assay

After parasite infections produced 60–70% lysis of the HFF monolayer in their respective flasks, *T. gondii ku80::hxgprt*, Δ*TgCBAP*, and the cosmid complemented clone (Δ*TgCBAP*::TOXOF64) were syringe-released and filtered with a 3 µm Nuclepore syringe filter (Whatman, GE Healthcare). 2×10^5^ parasites of each strain were transferred pairwise to a freshly confluent monolayer of HFF cells in a 25 cm^2^ flask. Immediately following complete lysis of the monolayers (ca. 36 h post-infection), 0.3 ml of parasites resuspended in culture supernatant were added to a new HFF monolayer. Culture medium was replaced 12 h after each passage. After passages 1, 2, 4, 8, and 12 a sample of extracellular parasites was removed and the relative number of TgCBAP(-) and TgCBAP(+) parasites determined by immunofluorescence using blot-affinity purified rabbit polyclonal anti-TgCBAP antibody and anti-IMC1. All parasites within randomly selected 60X fields were scored as either positive or negative for TgCBAP until 200 parasites were counted per replicate, per strain, for a total of 400 parasites counted per passage #, per experiment.

### Morphometric analysis

Parasites resuspended in HBSS supplemented with 10 mM HEPES, pH 7.2, were adhered to the bottom of 8-well chambered coverglasses (Nunc, Rochester NY) using BD Cell-Tak. Parasites were visualized by differential interference contrast (DIC) microscopy using a 100X PlanApo objective on Nikon Eclipse TE300 microscope. Images were captured using an iXon 885 EMCCD camera as described above. The length of both the long and short axes of 100 parasites per parasite line was determined using ImageJ v1.48.

## Results and Discussion

### Identification of TgCBAP

Previous work demonstrated direct binding of the *T. gondii* conoid extension inhibitor, Conoidin A, to a triad of proteins with apparent molecular weights of 25–30 kDa and isoelectric points of 6.5–7 [17]. Two of these three proteins were identified as isoforms of peroxiredoxin II (TgPRXII) and the third (TgCBAP) was annotated as a conserved hypothetical protein (GenBank accession number XM_002368777, ToxoDB ID TGME49_267500).

Homologs of *TgCBAP* are found only within the genomes of apicomplexan organisms, most of which contain a single copy of the gene, and they contain no recognizable functional domains or motifs that would provide insight into their biological function(s). Like TgPRXII, TgCBAP contains multiple cysteine residues which could potentially act as thiol nucleophiles and bind covalently to Conoidin A. Interestingly, TgCBAP was detected in the conoid-enriched fraction in a proteomic study of the *T. gondii* cytoskeleton [26]. These preliminary results prompted us to test whether TgCBAP was the biologically relevant target of Conoidin A and to begin to characterize the function(s) of this novel protein.

### Generation of Δ*TgCBAP* knockout parasites

**Figure 1:**
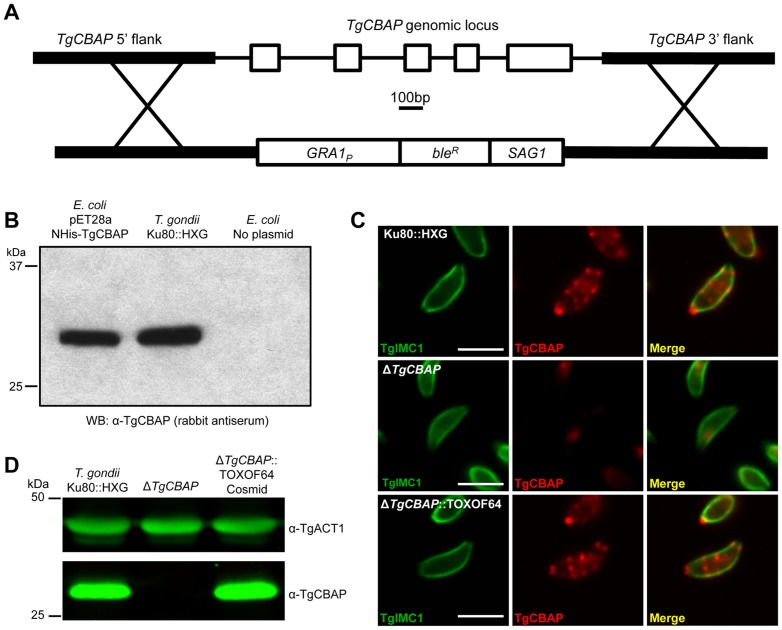
Generation of a Δ*TgCBAP* knockout parasite line. (**A**) The *TgCBAP* gene was ablated in *ku80::hxgprt* parasites using a construct encoding the *ble* gene, which encodes for resistance to the drug phleomycin [21], flanked by genomic sequences homologous to the 5′ and 3′ regions of the *TgCBAP* open reading frame. (**B**) Rabbit TgCBAP antiserum reacts with both the recombinant NHis-TgCBAP used as the antigen to generate the antiserum and with a single protein in *T. gondii* lysates with the predicted molecular weight of TgCBAP (25.7 kDa). (**C**) Immunofluorescence analysis of *T. gondii* parental (*ku80::hxgprt*), knockout (Δ*TgCBAP*), and cosmid complemented (Δ*TgCBAP*::TOXOF64) parasites labeled with anti-IMC1 (green) and blot affinity purified anti-TgCBAP (red) antibodies. Scale bar = 5 µm. (**D**) Lysates of *ku80::hxgprt*, Δ*TgCBAP*, and Δ*TgCBAP*::TOXOF64 parasite lines were analyzed by Western blot using antisera against TgCBAP and TgACT1 (loading control).

If TgCBAP is the relevant target of Conoidin A, then parasites lacking TgCBAP should show altered sensitivity to the compound. The genomic locus encoding *TgCBAP* was therefore ablated by transfection of a plasmid construct encoding a phleomycin resistance cassette [21] flanked by 5′ and 3′ sequences from upstream and downstream of the *TgCBAP* coding sequence (Figure 1A). We generated rabbit anti-TgCBAP antisera, which detected a single protein of the predicted molecular weight in *T. gondii* lysates (Figure 1B), and we used this antibody to screen the transfected parasite population for *ΔTgCBAP* knockouts. After two rounds of selection, ∼30% of the parasite population lacked TgCBAP by immunofluorescence. Parasites were cloned by limiting dilution and again screened for loss of TgCBAP expression by immunofluorescence (compare Figure 1C, top and middle panels). Western blotting confirmed the ablation of TgCBAP expression in several parasite clones, one of which (Figure 1D) was used for all subsequent experiments.

For phenotypic studies, the Δ*TgCBAP* parasites were then complemented by transfection with a 38.6 kb cosmid (TOXOF64) spanning the entire *TgCBAP* locus. After one round of selection with pyrimethamine, parasites were cloned by limiting dilution and screened for the presence of TgCBAP expression by Western blot using the TgCBAP antiserum. Expression of TgCBAP in the cosmid-complemented parasites was confirmed both by immunofluorescence and Western blotting (Figure 1C, lower panels, and Figure 1D).

### Parasites lacking TgCBAP show unchanged sensitivity to Conoidin A

**Figure 2:**
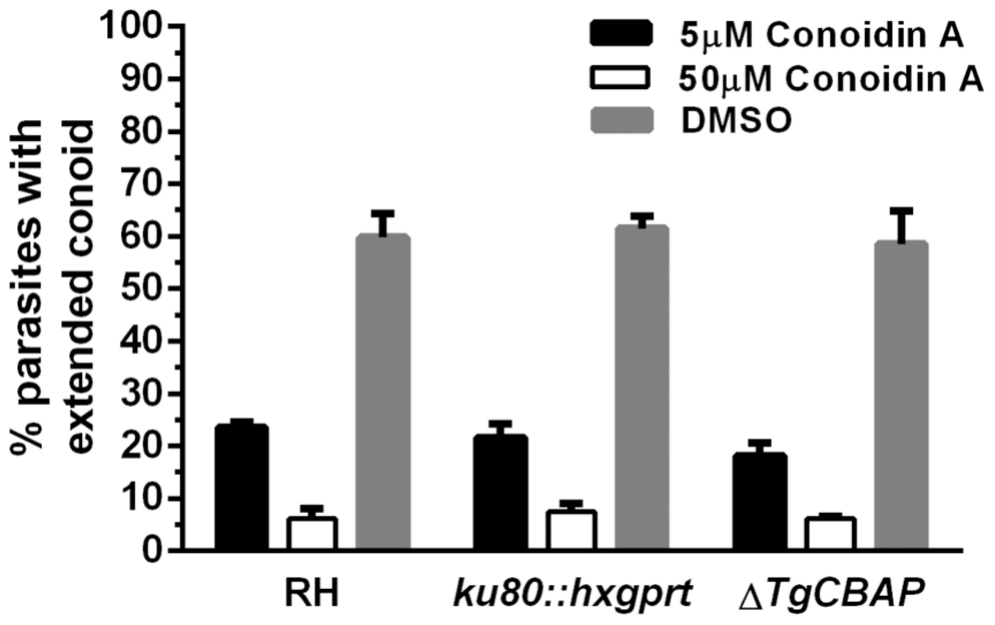
Δ*TgCBAP* knockout parasites display unaltered sensitivity to Conoidin A. Ionomycin-induced conoid extension assays using Δ*TgCBAP*, parental (*ku80::hxgprt*), and RH strain parasites. Parasites were pretreated for 15 min with either 5 µM or 50 µM Conoidin A (or an equivalent amount of DMSO), followed by a 5 min incubation in the presence of 10 µM ionomycin. Ionomycin-induced conoid extension is blocked to the same extent by Conoidin A in the three parasite lines. The data shown represent the mean values from 3 independent experiments (+/− SEM).

To test the hypothesis that TgCBAP is the biologically relevant target of Conoidin A, we compared the ability of Conoidin A to inhibit conoid extension in the Δ*TgCBAP* and parental *ku80::hxgprt* parasites. RH strain parasites were also included in these assays, since this was the original strain against which Conoidin A was shown to have an effect [16]. No statistically significant differences in the sensitivity of *ku80::hxgprt*, RH, or Δ*TgCBAP* parasites to Conoidin A were observed in conoid extension assays (Figure 2); the compound inhibited ionophore-induced conoid extension in all three parasite lines to an equivalent extent.

TgCBAP therefore does not appear to be required either for extension of the conoid or for inhibition of conoid extension by Conoidin A, even though TgCBAP binds directly to Conoidin A [17], is found in a conoid-enriched cytoskeletal fraction [26] and localizes to a ring-like structure at the apical end of the parasite (discussed further below). *T. gondii PRXII* knockout parasites also show unchanged sensitivity to Conoidin A. However, *T. gondii* expresses four different peroxiredoxins; if these were to serve overlapping functions, the disruption of any single *T. gondii PRX* gene might not be sufficient to confer resistance to the compound. We therefore cannot yet rule out a role for peroxiredoxin proteins in the mechanism of action of Conoidin A [17].

### TgCBAP localizes to three distinct subcompartments in *T. gondii* and associates with the cytoskeleton

**Figure 3:**
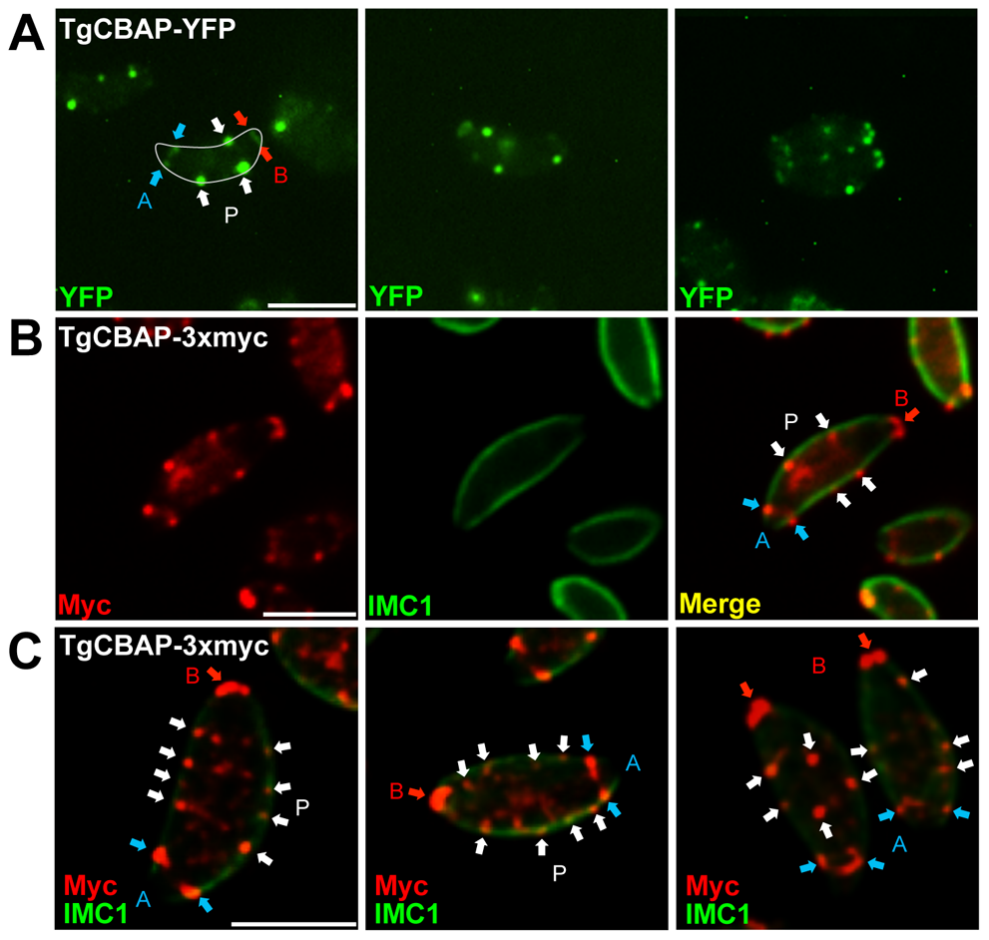
TgCBAP localizes to least three distinct subcompartments in *T. gondii* parasites. Immunofluorescence assays of *T. gondii ku80::hxgprt* parasites expressing C-terminal endogenously tagged TgCBAP-YFP or TgCBAP-3xmyc fusion proteins. (**A**) Three representative examples of live intracellular parasites expressing TgCBAP-YFP. The parasite in the left panel has been outlined in white for reference. The right panel shows two parasites that have just emerged from a mother cell. TgCBAP localizes to rings at the apical (“A”, blue arrows) and basal (“B”, red arrows) ends of the parasites as well as punctate structures along the periphery (“P”, white arrows). (**B**) Paraformaldehyde-fixed extracellular parasites expressing TgCBAP-3xmyc with anti-myc (red) and anti-IMC1 (green) antibodies show an identical staining pattern as TgCBAP-YFP. (**C**) Three representative deconvolved maximum intensity projections of TgCBAP-3xmyc parasites dual labeled for myc and IMC1 as in panel B. The peripheral punctate structures containing TgCBAP vary in number and location. A similar punctate labeling along the periphery was seen after fixation with 0.2% (v/v) glutaraldehyde (data not shown). Scale bars = 5 µm.

Immunofluorescence with affinity-purified anti-TgCBAP showed it had an intriguing distribution within the parasite, being found in both ring-like structures at the apical and basal ends of the parasite and distinct punctate structures distributed around the parasite periphery (Figure 1C). To confirm this localization and to generate tools for dual immunofluorescence and live-cell imaging, we fused the 3′ end of *TgCBAP* with either YFP or a 3xmyc epitope tag at the genomic locus in *ku80::hxgprt* parasites [18]. Both tagged proteins showed a localization indistinguishable from that seen with the anti-TgCBAP antibody: small, ring-like structures near the apical and basal ends of the parasite as well as punctate spots around the parasite periphery (Figure 3). The clearest pattern in terms of signal-to-background was obtained by immunofluorescence using the TgCBAP-3xmyc parasites (Figure 3B and C). The IMC1 signal extends past the apical TgCBAP ring (Figures 3B and C), distinguishing the TgCBAP ring from the apical polar ring. Maximum intensity projections of deconvolved images of TgCBAP-3xmyc parasites clearly show heterogeneous punctate spots around the periphery of the parasite (Figure 3C). However, in many parasites, the spots appear to be organized in rings around the circumference of the parasite (Figure 3C & Movies S1 & S2).

**Figure 4:**
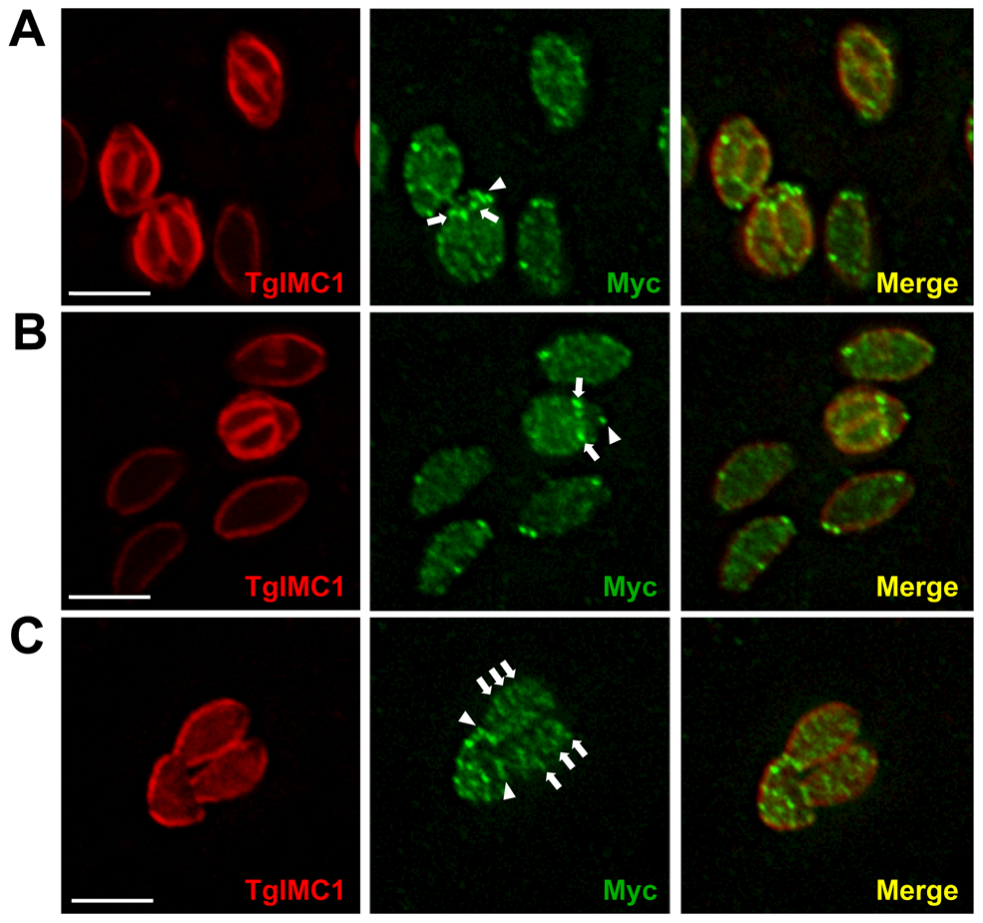
TgCBAP localizes to forming daughter parasites during endodiogeny. Immunofluorescence localization of endogenously tagged TgCBAP-3xmyc within *T. gondii ku80::hxgprt* parasites undergoing endodiogeny. Parasites were stained with anti-IMC1 (red) to outline the mother and daughter parasites and anti-myc (green) to localize TgCBAP-3xmyc; deconvolved maximum intensity projections are shown. (**A,B**) A basal ring of TgCBAP staining is evident in both the mother cell (arrowhead) and forming daughters (arrows). The peripheral puncta are difficult to resolve on the surface of the forming daughter cells; however, 3D reconstructions suggest that these may be present as well (see Movie S1) (**C**) Peripheral puncta organized in stripes are clearly evident in mature, emerging daughter cells (arrows), as are the basal rings (arrowheads). Scale bars = 5 µm.

During endodiogeny, the process by which daughter cells are produced within a mother cell, the apical and basal TgCBAP rings are also seen in daughters as they form within the mother cell (Figures 4A and B and Movie S1). Rings of peripheral spots are difficult to visualize on the forming daughters, although 3D reconstructions suggest their presence (Movie S1) and they are clearly present by the time the daughter parasites emerge from the mother cell (Figure 4C). The nature of the ring-like compartment(s) around the parasite periphery is unknown but may in some way relate to the junctions between the plates of the IMC [7].

**Figure 5:**
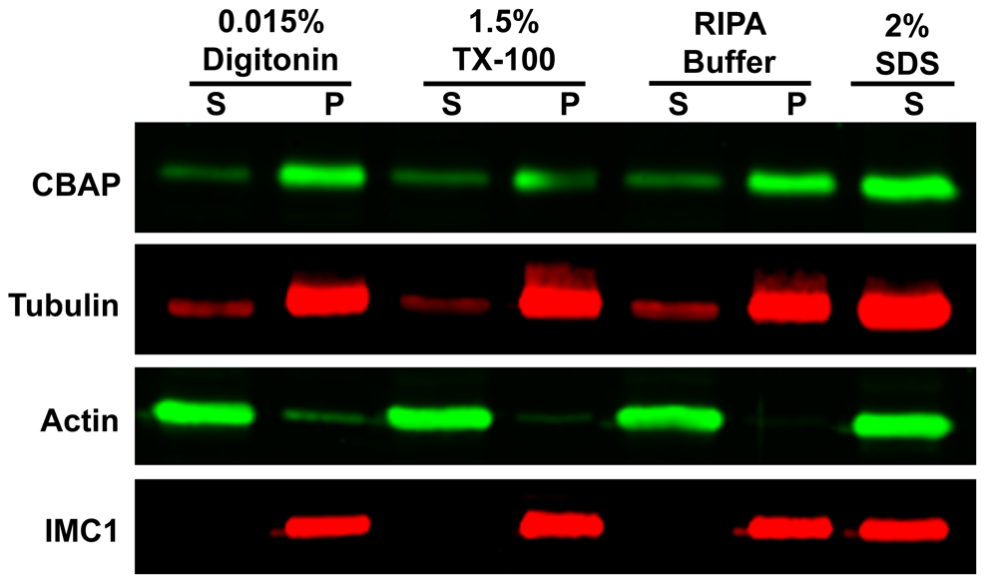
The detergent extraction profile of TgCBAP suggests association with the parasite cytoskeleton. Parasites were extracted with four different detergent-containing buffers (see Methods), centrifuged at 21,000*xg*, and analyzed by Western blot using antibodies against TgCBAP, TgTUB1, TgACT1, and TgIMC1. P, pellet; S, supernatant.

The small apical and basal ring structures suggest an association of TgCBAP with the parasite cytoskeleton, as previously reported [26]. The results of differential detergent extraction are consistent with a cytoskeletal association (Figure 5): the extraction profile of TgCBAP is identical to that of tubulin and IMC1, and different from that of actin, which is present primarily in a nonpolymerized form in *T. gondii* [27].

### TgCBAP partially co-localizes with TgMORN1 and TgISP1

**Figure 6:**
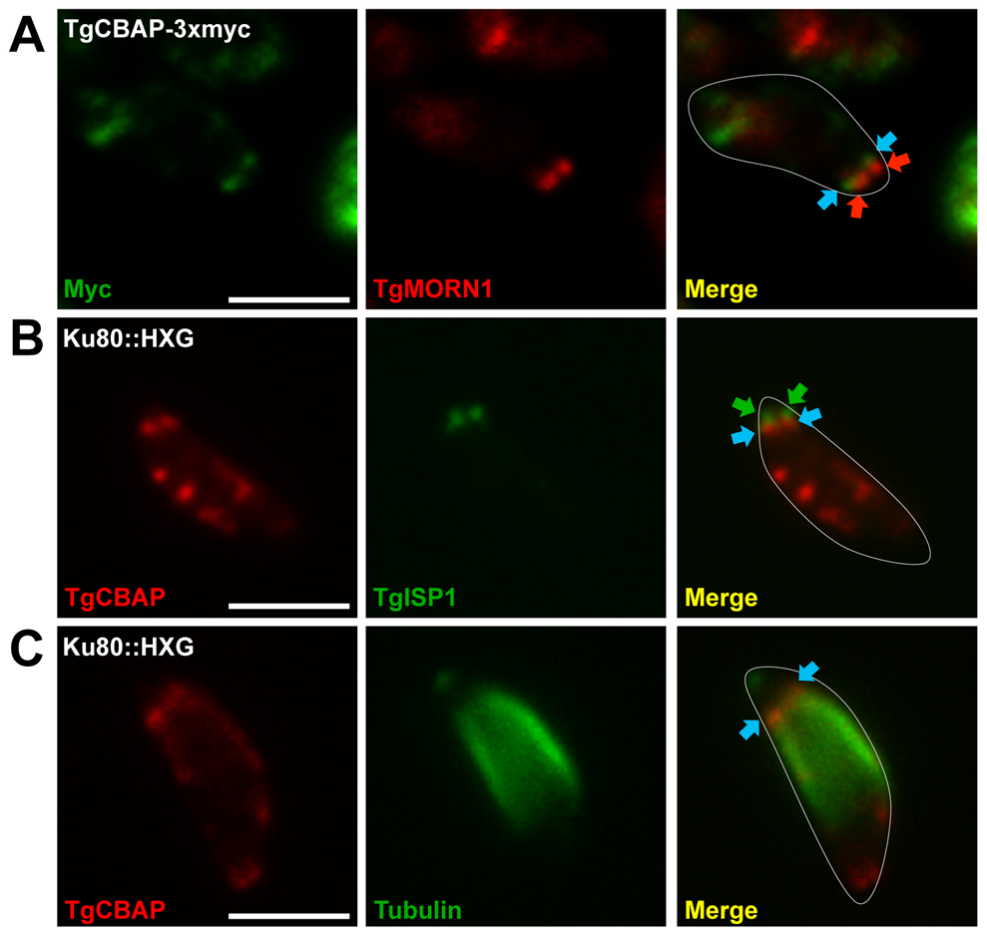
TgCBAP localization relative to the cytoskeletal proteins TgMORN1 and TgISP1. (**A**) TgCBAP-3myc (blue arrowheads) localizes to a ring-like structure slightly anterior to TgMORN1 (red arrowheads) at the basal end of extracellular parasites. (**B**) The apical TgCBAP ring, labeled here with affinity-purified anti-TgCBAP (blue arrowheads), localizes slightly posterior to TgISP1 (green arrowheads). (**C**) The TgCBAP apical ring (blue arrowheads) is also located at the anterior edge of the subpellicular microtubules. Parasites in (A) and (B) were extracted in 0.5 mM deoxycholate; parasites in (C) were extracted in 10 mM deoxycholate. Labeling of the peripheral puncta is disrupted by deoxycholate extraction. The parasites in the merged images have been outlined in white for reference. Scale bars = 5 µm.

To better define the spatial distribution of TgCBAP relative to previously described cytoskeletal markers, dual label immunofluorescence assays were performed with TgCBAP and the basal marker TgMORN1 [23], the apical marker TgISP1 [24], or tubulin [15]. The basal TgCBAP ring-like structure is found slightly anterior to TgMORN1 (Figure 6A), and at the apical end of the parasite, the TgCBAP ring localizes slightly posterior to TgISP1 (Figure 6B), at the posterior end of a gap in tubulin staining between the conoid and subpellicular microtubules (Figure 6C). The gap in tubulin staining is likely an artifact caused by inaccessibility of the tubulin epitope in this region of the parasite [15].

### The localizations of TgMORN1 and TgISP1 are unaffected in parasites lacking TgCBAP

**Figure 7:**
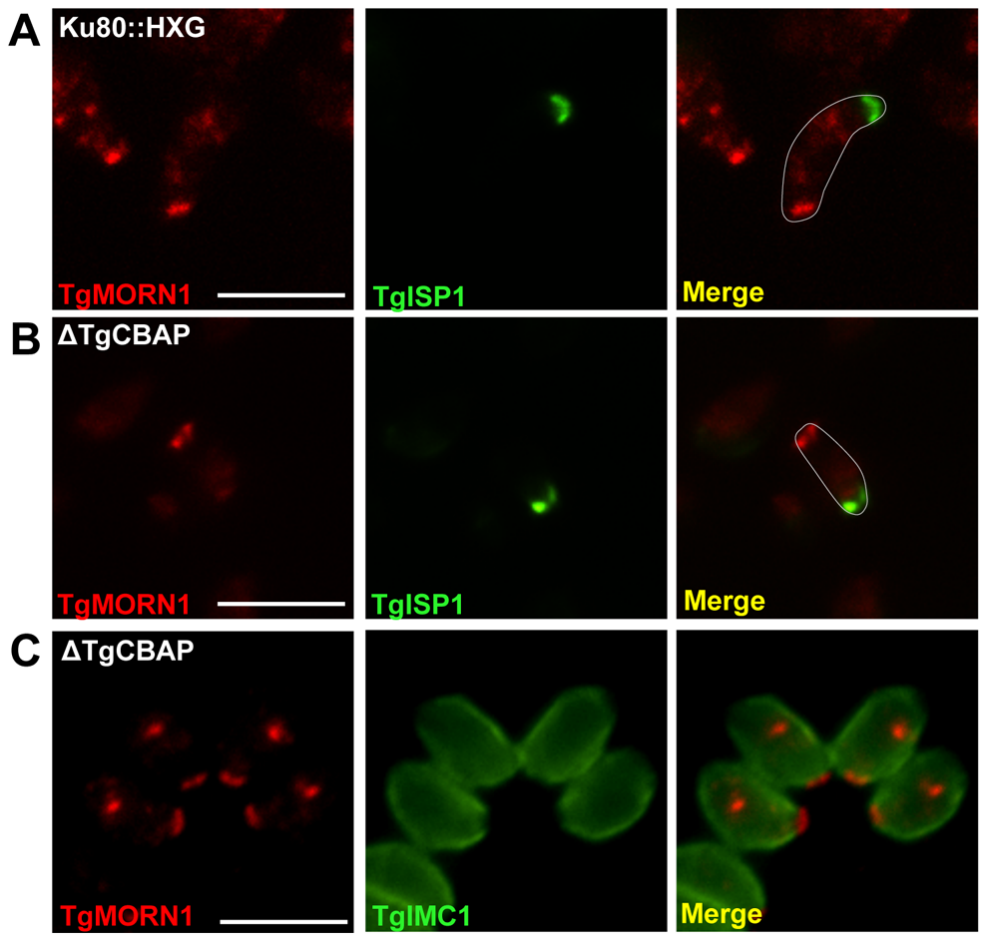
Disruption of TgCBAP does not affect the localization of TgMORN1 or TgISP1. TgMORN1 and TgISP1 localization in (**A**) parental (*ku80::hxgprt*) and (**B**) Δ*TgCBAP* parasites is indistinguishable. (**C**) Disruption of TgCBAP also does not change the rosette-like organization of parasites within the parasitophorous vacuole. The parasites in (A) and (B) have been outlined in white in the merged images for reference. Scale bars = 5 µm.

Given the proximity of TgCBAP to TgMORN1 and TgISP1, we investigated whether the localization of these proteins is altered in the Δ*TgCBAP* parasites and found that it was not (Figure 7). Furthermore, and in contrast to TgMORN1 [28], [29], the lack of TgCBAP had no effect on the organization of parasites within the parasitophorous vacuole (Figure 7C). A direct functional interaction between TgCBAP and TgMORN1 therefore seems unlikely, consistent with the fact that TgCBAP was not identified in a proteomic analysis of the TgMORN1 complex [30].

### Effect of TgCBAP depletion on parasite morphology and growth

**Figure 8:**
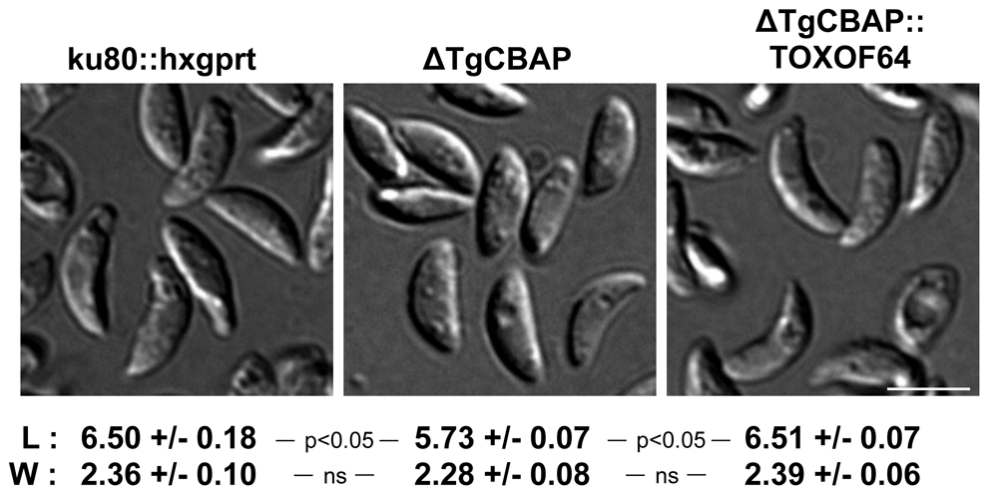
Parasites lacking TgCBAP are significantly smaller than parental parasites. Length (L) and width (W) dimensions of *T. gondii* parental (*ku80::hxgprt*), knockout (Δ*TgCBAP*), and cosmid-complemented (Δ*TgCBAP*::TOXOF64) parasites were measured from DIC images of live extracellular parasites adhered to coverslips using BD Cell-Tak. The greatest dimension along the long and short axes of the parasite was determined using ImageJ and is shown in µm ± SEM. Parasites deficient in TgCBAP are shorter than parental and cosmid-complemented parasites: length differences were statistically significant between *ku80::hxgprt* and Δ*TgCBAP* parasites and between Δ*TgCBAP* and Δ*TgCBAP*::TOXOF64 parasites, but not between *ku80::hxgprt* and Δ*TgCBAP*::TOXOF64 parasites (unpaired Student's t-test; ns  =  not significant). Scale bars = 5 µm.

Previous work from our laboratory showed that parasites lacking another cytoskeletal protein (TgPhIL1) that forms rings at the apical end of the parasite were significantly shorter and wider than parental parasites [31]. Δ*TgCBAP* parasites are also significantly shorter than wild-type parasites (Figure 8), but their width remains unchanged. Complementation of Δ*TgCBAP* parasites with cosmid TOXOF64 containing wild-type *TgCBAP* restored the morphology to parental dimensions (Figure 8).

**Figure 9:**
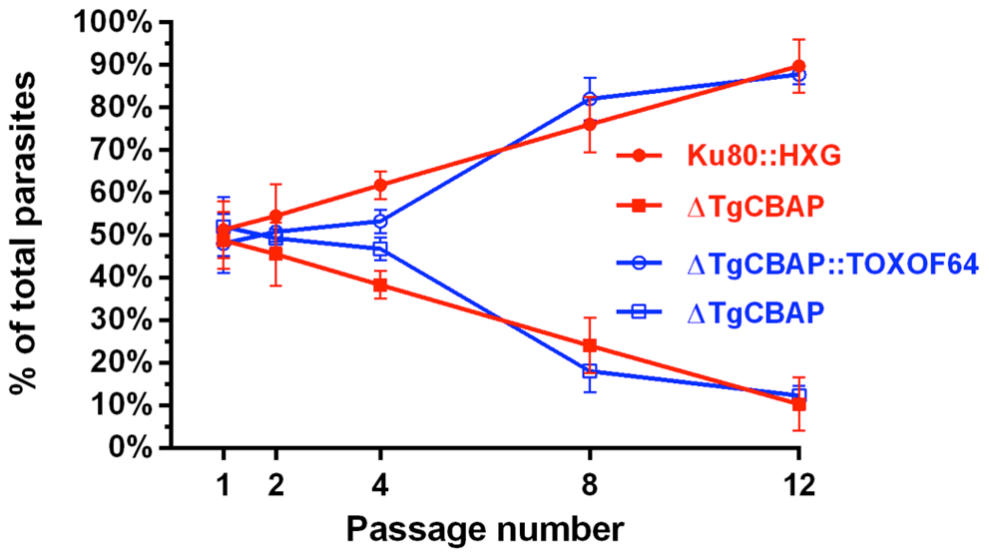
Growth defect of Δ*TgCBAP* knockout parasites is restored in cosmid-complemented parasites. Equal numbers of *T. gondii* parental (*ku80::hxgprt*), knockout (Δ*TgCBAP*), and cosmid-complemented (Δ*TgCBAP*::TOXOF64) parasites were added pairwise to HFF monolayers and serially passaged. At passage number 1, 2, 4, 8, and 12, immunofluorescence detection of TgCBAP was performed on extracellular parasites. Each experiment was performed in duplicate and results shown are the averages of the two biological replicates (+/− SEM). Parental (*ku80::hxgprt*; red closed circles) and cosmid-complemented (Δ*TgCBAP*::TOXOF64; blue open circles) parasites exhibited a faster growth than parasites lacking TgCBAP (Δ*TgCBAP*; red closed squares or blue open squares). The difference between parasites expressing TgCBAP and parasites lacking TgCBAP is significant (unpaired Student's t-test, p<0.05) by passage 8.

During routine passage, it was apparent that Δ*TgCBAP* parasites required more time to completely lyse host monolayers than the parental line. Growth competition assays revealed that Δ*TgCBAP* parasites do indeed display a significant growth impairment compared to both parental *ku80::hxgprt* and complemented Δ*TgCBAP*::TOXOF64 parasites (Figure 9).

## Conclusions

TgCBAP is a novel protein of *T. gondii* that localizes to three distinct subcompartments within the parasite and appears to be associated with the cytoskeleton. The precise nature of the three subcompartments and the proteins with which TgCBAP interacts will be the subject of future investigation. Although the lack of recognizable functional motifs or domains precludes immediate insight into the function of TgCBAP or its homologs in other apicomplexan parasites, *T. gondii* tachyzoites lacking TgCBAP are smaller than the parasites from which they were derived and show a growth defect in culture. TgCBAP therefore appears to play an as yet undefined role in maintaining parasite size, which in turn may be important for parasite fitness.

## Supporting Information

Movie S1 **3D reconstruction of TgCBAP localization.** Reconstruction of deconvolved images of extracellular parasites rendered as a 3D movie. Parasites expressing TgCBAP-3xmyc were stained for IMC1 (green) and myc (red). Scale bar = 10 µm. Note the two parasites in the process of endodiogeny in the upper left quadrant.
(MPG)

Movie S2 **3D reconstruction of TgCBAP localization.** Reconstruction of deconvolved images of extracellular parasites rendered as a 3D movie. Parasites expressing TgCBAP-3xmyc were stained for IMC1 (green) and myc (red). Scale bar = 10 µm.
(MPG)
